# Chondrocyte-targeted exosome-mediated delivery of Nrf2 alleviates cartilaginous endplate degeneration by modulating mitochondrial fission

**DOI:** 10.1186/s12951-024-02517-1

**Published:** 2024-05-24

**Authors:** Zhidi Lin, Guangyu Xu, Xiao Lu, Siyang Liu, Fei Zou, Xiaosheng Ma, Jianyuan Jiang, Hongli Wang, Jian Song

**Affiliations:** Department of Orthopedics, Huashan Hospital, Fudan University, Shanghai, 200040 China

**Keywords:** Cartilaginous endplate degeneration, Engineered exosome, Nrf2, Drp1, Mitochondrial fission, Apoptosis

## Abstract

**Background:**

Cartilaginous endplate (CEP) degeneration, which is an important contributor to intervertebral disc degeneration (IVDD), is characterized by chondrocyte death. Accumulating evidence has revealed that dynamin-related protein 1 (Drp1)-mediated mitochondrial fission and dysfunction lead to apoptosis during CEP degeneration and IVDD. Exosomes are promising agents for the treatment of many diseases, including osteoporosis, osteosarcoma, osteoarthritis and IVDD. Despite their major success in drug delivery, the full potential of exosomes remains untapped.

**Materials and methods:**

In vitro and in vivo models of CEP degeneration were established by using lipopolysaccharide (LPS). We designed genetically engineered exosomes (CAP-Nrf2-Exos) expressing chondrocyte-affinity peptide (CAP) on the surface and carrying the antioxidant transcription factor nuclear factor E2-related factor 2 (Nrf2). The affinity between CAP-Nrf2-Exos and CEP was evaluated by in vitro internalization assays and in vivo imaging assays. qRT‒PCR, Western blotting and immunofluorescence assays were performed to examine the expression level of Nrf2 and the subcellular localization of Nrf2 and Drp1. Mitochondrial function was measured by the JC-1 probe and MitoSOX Red. Mitochondrial morphology was visualized by MitoTracker staining and transmission electron microscopy (TEM). After subendplate injection of the engineered exosomes, the degree of CEP degeneration and IVDD was validated radiologically and histologically.

**Results:**

We found that the cargo delivery efficiency of exosomes after cargo packaging was increased by surface modification. CAP-Nrf2-Exos facilitated chondrocyte-targeted delivery of Nrf2 and activated the endogenous antioxidant defence system in CEP cells. The engineered exosomes inhibited Drp1 S616 phosphorylation and mitochondrial translocation, thereby preventing mitochondrial fragmentation and dysfunction. LPS-induced CEP cell apoptosis was alleviated by CAP-Nrf2-Exo treatment. In a rat model of CEP degeneration, the engineered exosomes successfully attenuated CEP degeneration and IVDD and exhibited better repair capacity than natural exosomes.

**Conclusion:**

Collectively, our findings showed that exosome-mediated chondrocyte-targeted delivery of Nrf2 was an effective strategy for treating CEP degeneration.

**Graphic abstract CAP-Nrf2-Exos delivered Nrf2 into CEP cells and alleviated LPS-induced apoptosis by inhibiting Drp1-mediated mitochondrial fission:**

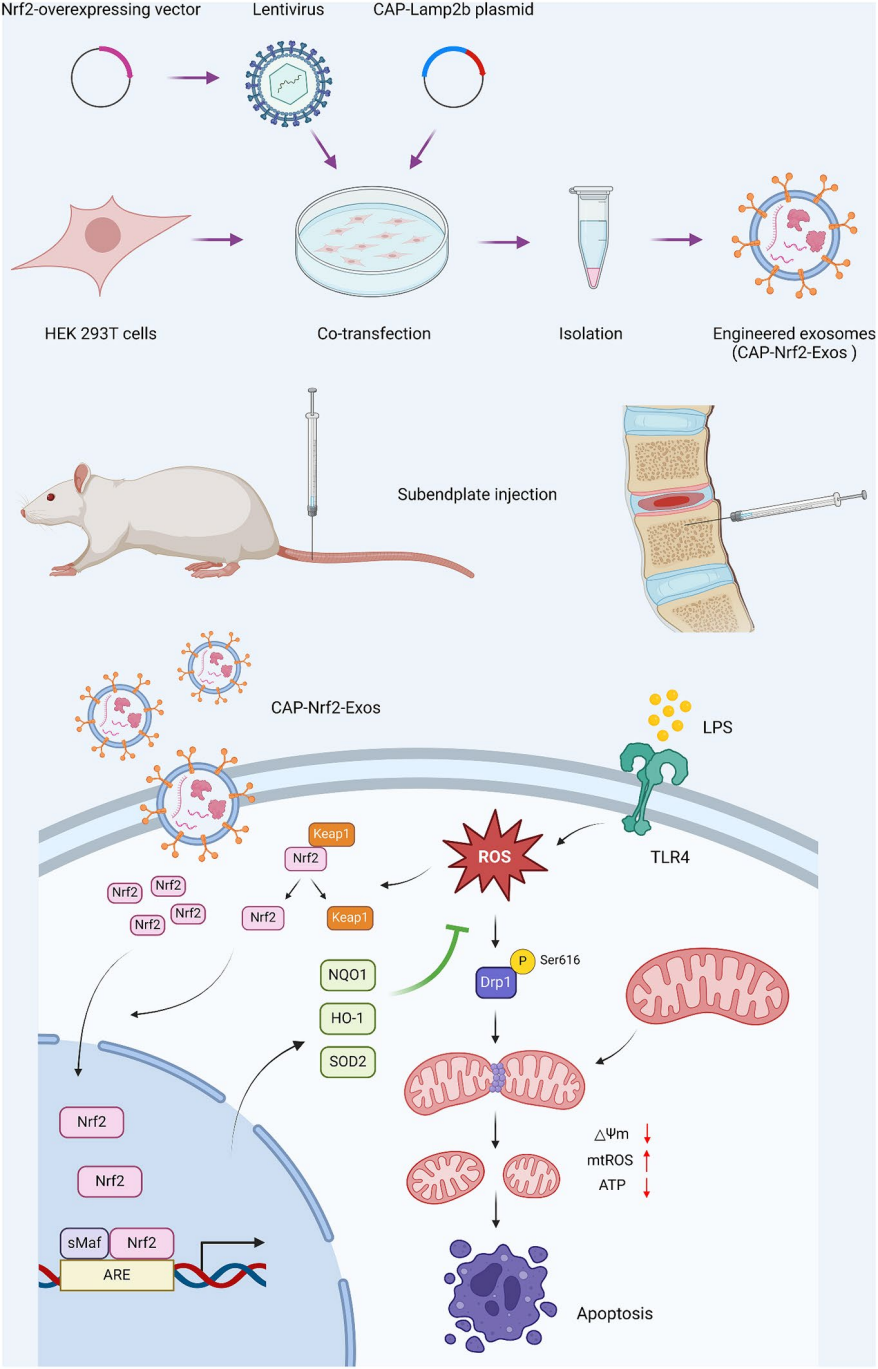

## Introduction

Modic changes (MCs) are commonly observed by magnetic resonance imaging (MRI) of degenerative spinal diseases and are closely related to cartilaginous endplate (CEP) degeneration and discogenic low back pain [1]. The prevalence of MCs is approximately 0.5-47.1% in the asymptomatic population and 8-80.1% in patients with low back pain [2]. CEP degeneration is a key initiator of intervertebral disc degeneration (IVDD) [3]. However, the CEP is a neglected structure in IVDD research, and most studies have focused on the nucleus pulposus (NP). The CEP is a unique tissue type that differs from articular cartilage [4]. The CEP is a thin layer of hyaline cartilage positioned between the vertebral body and the NP that acts as a physical barrier to prevent NP bulging and a channel to provide oxygen, glucose and other nutrients [5]. The intervertebral disc (IVD) is considered the largest avascular organ in the body. The NP and the inner layer of the annulus fibrosus (AF) mainly rely on the passive diffusion of nutrients through the CEP [6]. Chondrocyte apoptosis results in insufficient nutrient supply in the disc and accelerates the development of IVDD [7].

Mitochondrial dysfunction and dynamics play vital roles in IVDD [8]. Mitochondria, which are bound by a double membrane, are central regulators of energy metabolism, calcium signalling, reactive oxygen species (ROS) production and cell fate decisions [9]. Since mitochondria are highly dynamic organelles, they constantly undergo fission and fusion, which is termed mitochondrial dynamics. The GTPase dynamin-related protein 1 (Drp1) mediates mitochondrial fission by forming helical oligomers that wrap around the mitochondrial outer membrane and scission it [10]. Fission results in the production of new mitochondria during cell division, enabling the redistribution of mitochondria and facilitating the segregation of damaged mitochondria. In contrast, fusion mediates the exchange of intramitochondrial contents among different mitochondria. A balance between fission and fusion is critical for maintaining mitochondrial homeostasis in response to different stresses [11]. Disruption of mitochondrial dynamics triggers the accumulation of defective mitochondria with abnormal morphology and ultimately leads to cell death [12]. Nuclear factor E2-related factor 2 (Nrf2) is a master transcription factor that regulates the endogenous antioxidant defence system. Accumulating evidence has revealed that Nrf2 protects disc cells from oxidative stress damage during IVDD progression [13]. Under physiological conditions, Nrf2 is negatively regulated by Kelch-like ECH-associated protein 1 (Keap1) via ubiquitination and proteasome-dependent degradation. In response to stress, Keap1 undergoes conformational alterations and dissociates from Nrf2. Subsequently, Nrf2 translocates into the nucleus, binds to the antioxidant response element (ARE) by forming heterodimers with small musculoaponeurotic fibrosarcoma (MAF) proteins (sMAFs) and promotes the expression of multiple downstream antioxidant genes, including NADP(H) quinone oxidoreductase 1 (NQO1), haem-oxygenase-1 (HO-1) and superoxide dismutase 2 (SOD2) [14]. Moreover, Nrf2 is associated with the inhibition of Drp1 activity, mitochondrial fission and dysfunction [15]. The Nrf2-Drp1 axis also participates in the regulation of CEP degeneration and IVDD [16].

Exosomes are naturally produced nanoscale vesicles (30–150 nm in diameter) enclosed by a lipid membrane bilayer that are secreted by nearly all cell types [17]. The released exosomes carry nucleotides, proteins and lipids. These factors act as intercellular messengers by transferring cargo from donor cells to recipient cells [18]. Exosomes, especially those derived from mesenchymal stem cells, can suppress inflammation, oxidative stress, neovascularization, apoptosis, pyroptosis and ferroptosis and improve cell proliferation, differentiation and ECM homeostasis [19, 20]. Therefore, exosomes can delay the progression of IVDD through multiple mechanisms. Previously, our team reported that exosomes derived from bone marrow mesenchymal stem cells (BMSC-Exos) could inhibit apoptosis and inflammation in NP cells by activating the Keap1/Nrf2 pathway [21]. The use of exosomes is a promising alternative to traditional cell therapy because the harsh microenvironment in degenerated IVDs limits the survival rate of transplanted cells [22]. Although exosome-based therapy has shown excellent performance in treating IVDD, its full potential had not yet been revealed [23].

Exosomes are highly engineerable. Surface modification and cargo packaging are effective strategies for generating engineered exosomes [24]. Genetic engineering is a convenient technique for creating exosomes that selectively target specific recipient cells to enhance therapeutic effects. Several membrane proteins, such as CD9, CD63, CD81 and Lamp2b, are universally expressed on exosomes. Cell-targeted peptides can be displayed on the surface of exosomes through genetic fusion to the extracellular domains of these proteins [25]. For example, Alvarez-Erviti et al. [26] fused Lamp2b to a neuron-specific RVG peptide to construct the RVG-Lamp2b plasmid and transfected dendritic cells. In vitro and in vivo assays showed that dendritic cell-derived exosomes specifically delivered their cargo to neuronal cells and brain tissue, validating their targeting capacity. The methods for encapsulating cargo within exosomes can be roughly divided into endogenous and exogenous loading methods. For endogenous involve genetically engineering donor cells to overexpress specific DNA, noncoding RNAs, mRNAs or proteins, which are subsequently incorporated into donor cell-derived exosomes [27]. Mizrak et al. [28] first reported that extracellular vesicles (EVs) could deliver therapeutic mRNAs/proteins to treat cancer. They developed genetically engineered EVs by transfecting a plasmid overexpressing the prodrug-converting enzyme cytosine deaminase (CD)-uracil phosphoribosyltransferase (UPRT) into HEK293T cells and isolated EVs loaded with CD-UPRT mRNA and protein. CD-UPRT mRNA and protein were abundant and functional in the engineered EVs.

In the present study, we designed, generated, and preclinically evaluated the translational potential of chondrocyte-targeted exosomes enriched in Nrf2 as a novel strategy for alleviating the progression of CEP degeneration.

## Results

### Preparation of engineered exosomes

**Fig. 1:**
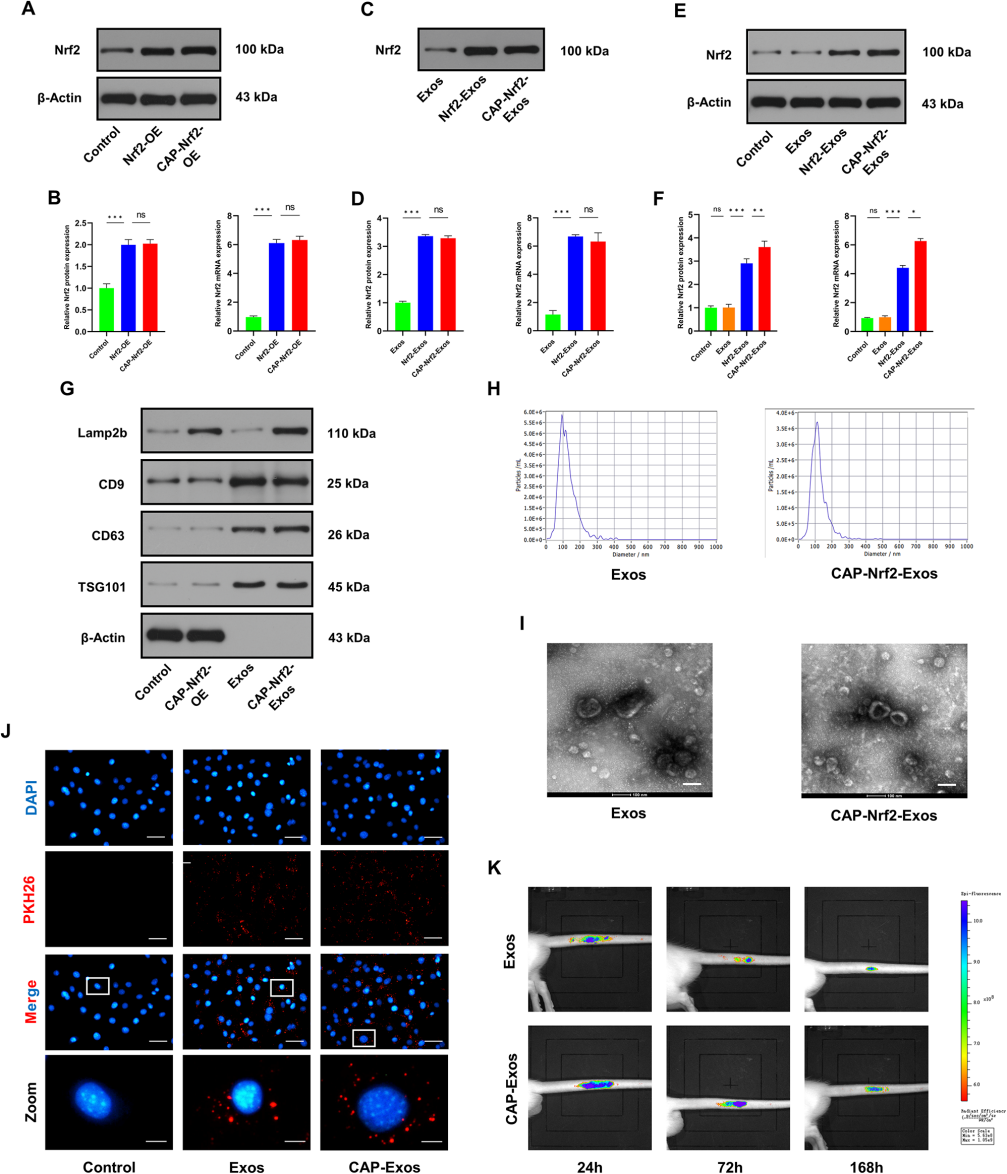
Construction and features of engineered CAP-Nrf2-Exos. (**A, B**) Western blot and qRT‒PCR analysis showing the mRNA and protein expression levels of Nrf2 in HEK293T cells. (**C, D**) Western blot and qRT‒PCR analysis of Nrf2 in the engineered exosomes. (**E, F**) Western blot and qRT‒PCR analysis of Nrf2 expression in CEP cells after treatment with engineered exosomes. (**G**) Western blot analysis of Lamp2b, CD9, CD63, TSG101, and β-Actin in HEK293T cells, HEK293T cells transfected with CAP-Nrf2 overexpression plasmids, Exos and CAP-Nrf2-Exos. (**H**) Exos and CAP-Nrf2-Exos were examined by NTA. (**I**) TEM analysis of Exos and CAP-Nrf2-Exos isolated from the culture medium of HEK293T cells. Scale bar, 100 nm. (**J**) Fluorescence images of PKH26-labelled Exos and CAP-Exos internalized by CEP cells. Scale bar, 50 μm and 10 μm. (**K**) Fluorescence images of DiR-labelled Exos and CAP-Exos in the CEP and IVD in the groups mentioned above. (*n* = 3, *p < 0.05; **p < 0.01; ***p < 0.001)

We previously showed that bone marrow mesenchymal stem cell-derived exosomes (BMSC-Exos) could ameliorate intervertebral disc degeneration (IVDD) by promoting Nrf2 expression and nuclear translocation, thereby increasing antioxidant responses and scavenging excessive reactive oxygen species (ROS) in nucleus pulposus (NP) cells [21]. Exosomes enriched with Nrf2 may exert cytoprotective effects. Thus, an Nrf2-overexpressing (Nrf2-OE) lentiviral vector was constructed. To improve the affinity between the engineered exosomes and cartilaginous endplate (CEP) cells, a chondrocyte-affinity peptide (CAP, DWRVIIPPRPSA) was fused to the N-terminus of lysosomal-associated membrane protein 2b (Lamp2b) [29]. Then, the CAP-Lamp2b plasmid and the Nrf2-OE lentivirus were cotransfected into HEK293T cells (CAP-Nrf2-OE). The mRNA and protein expression of Nrf2 was upregulated in HEK293T cells transfected with the Nrf2-OE vector alone or cotransfected with the Nrf2 expression vector and the CAP-Lamp2b plasmid (CAP-Nrf2-OE) (Fig. 1A, B). Exosomes were isolated from the culture medium by ultracentrifugation.

### Characterization of engineered exosomes

To evaluate the properties of the engineered exosomes, we collected exosomes derived from control HEK293T cells (Exos), Nrf2-overexpressing HEK293T cells (Nrf2-Exos), and HEK293T cells transfected with the CAP-Lamp2b plasmid (CAP-Exos), and HEK293T cells cotransfected with Nrf2 and the CAP-Lamp2b plasmid (CAP-Nrf2-Exos). qRT‒PCR and Western blot analysis revealed that Nrf2 mRNA and protein were robustly expressed in Nrf2-Exos and CAP-Nrf2-Exos (Fig. 1C, D), suggesting that Nrf2 was encapsulated in the engineered exosomes. To investigate the delivery potential of the engineered exosomes, we treated CEP cells with the different exosomes. Nrf2-Exos increased Nrf2 mRNA and protein levels in CEP cells. Moreover, the expression level of Nrf2 was further increased in CEP cells treated with CAP-Nrf2-Exos, indicating that the engineered exosomes could efficiently enter CEP cells via CAP (Fig. 1E, F). CAP-Nrf2-Exos and Exos were examined by transmission electron microscopy (TEM), nanoparticle tracking analysis (NTA) and Western blot analysis. Exosomal morphology and size were analysed and showed that CAP-Nrf2-Exos were typical saucer-shaped vesicles with a peak diameter of 110.6 nm, similar to the control exosomes. Western blot analysis of characteristic exosome membrane proteins, including Lamp2b, CD9, CD63, and TSG101, further verified the identity of CAP-Nrf2-Exos and Exos (Fig. 1G-I).

### Chondrocyte targeting of engineered exosomes

The in vitro uptake of PKH26-labelled CAP-Exos was confirmed by fluorescence microscopy. As shown in Fig. 1J, PKH26-labelled CAP-Exos were more efficiently internalized by CEP cells than control exosomes after 24 h. To explore whether the engineering strategy increased the retention of exosomes in vivo, DiR was used to label Exos and CAP-Exos. DiR fluorescence was examined by an in vivo imaging system (IVIS). Sprague‒Dawley (SD) rats were administered subendplate injections of Exos or CAP-Exos. The fluorescence intensity was higher in the CAP-Exo group than in the Exo group at each time point (24 h, 72 h and 168 h) (Fig. 1K). These findings revealed that CAP-Exos were better preserved and could target CEP in vivo, which was consistent with the in vitro data.

### CAP-Nrf2-Exos delivered Nrf2 into CEP cells and enhanced antioxidant responses

**Fig. 2:**
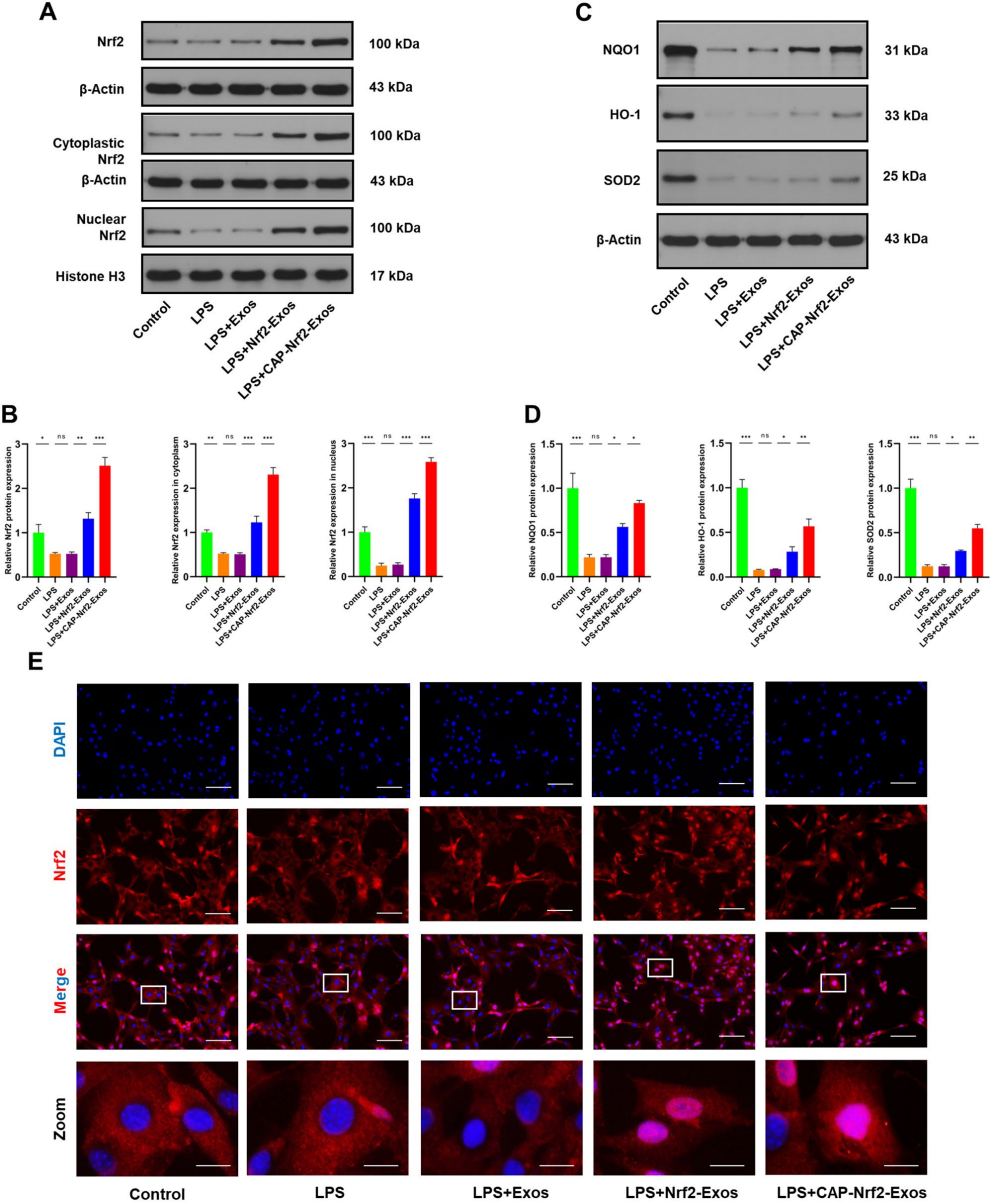
CAP-Nrf2-Exos delivered Nrf2 and activated antioxidant responses in CEP cells. (**A, B**) Western blot analysis and quantification of total Nrf2, cytoplasmic Nrf2 and nuclear Nrf2 in CEP cells after treatment with LPS and engineered exosomes. (**C, D**) Western blot analysis and quantification of NQO1, HO-1 and SOD2 in CEP cells after treatment with LPS and engineered exosomes. (**E**) Immunofluorescence staining of Nrf2 and DAPI in CEP cells in the groups mentioned above. Scale bar, 100 μm and 20 μm. (*n* = 3, *p < 0.05; **p < 0.01; ***p < 0.001)

To investigate the functions of the engineered exosomes, lipopolysaccharide (LPS) was used to trigger inflammation and CEP degeneration [30]. Western blot analysis revealed that the expression and activity of Nrf2 were decreased in LPS-treated CEP cells, and NADP(H) quinone oxidoreductase 1 (NQO1), haem-oxygenase-1 (HO-1) and superoxide dismutase 2 (SOD2) levels were decreased, indicating exhaustion of the endogenous antioxidant defence system. We next treated CEP cells with control exosomes and engineered exosomes under inflammatory conditions. Control exosomes did not alter Nrf2 expression or activity. In contrast, Western blot analysis showed that Nrf2-Exos and CAP-Nrf2-Exos increased Nrf2 protein levels and promoted the nuclear translocation of Nrf2 (Fig. 2A-D). Immunofluorescence staining further verified the colocalization of Nrf2 (red fluorescence) and DAPI (blue fluorescence) in LPS-treated CEP cells (Fig. 2E). Moreover, CAP-Nrf2-Exos could deliver more Nrf2 into CEP cells and trigger higher expression of antioxidant defence genes than Nrf2-Exos, validating the efficacy of this chondrocyte-targeted strategy.

### CAP-Nrf2-Exos inhibited Drp1-mediated mitochondrial fission in CEP cells

**Fig. 3:**
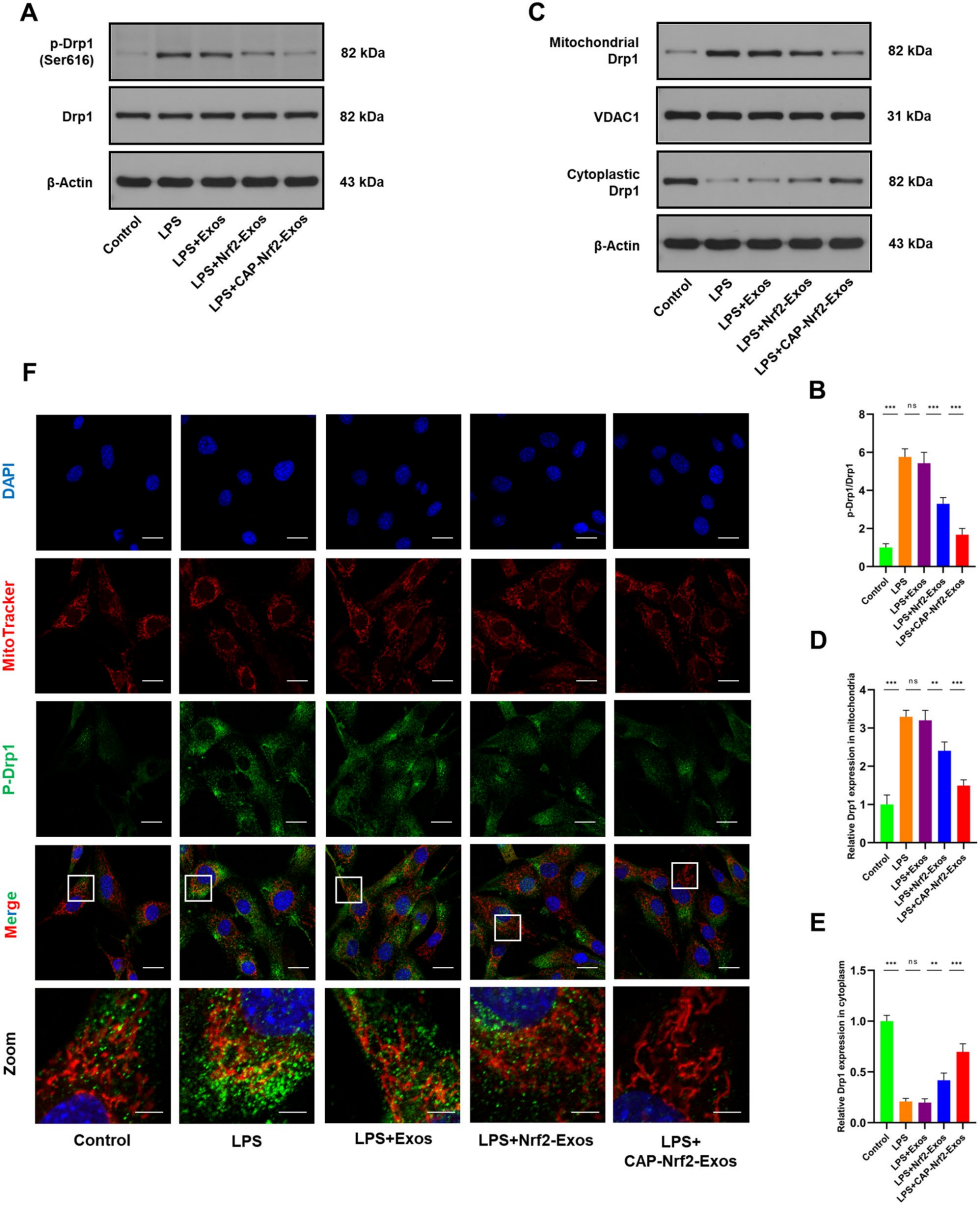
CAP-Nrf2-Exos inhibited Drp1-mediated mitochondrial fission. (**A, B**) Western blot analysis and quantification of p-Drp1 and Drp1 in CEP cells after treatment with LPS and engineered exosomes. (**C-E**) Western blot analysis and quantification of cytoplastic Drp1 and mitochondrial Drp1 in CEP cells after treatment with LPS and engineered exosomes. (**F**) MitoTracker Red staining and immunofluorescence staining of p-Drp1 and CEP cells after treatment with LPS and engineered exosomes. Scale bar, 20 μm and 5 μm (*n* = 3, *p < 0.05; **p < 0.01; ***p < 0.001)

We next explored the mechanisms by which Nrf2 signalling was enhanced by Nrf2-Exos and CAP-Nrf2-Exos. In addition to inflammatory responses, LPS exposure increases ROS production and triggers oxidative stress in CEP cells [31]. Inflammation and oxidative stress can induce Drp1 activation and mitochondrial fission and dysfunction [8]. Liu et al. reported that Nrf2 signalling protected against CEP degeneration [16] and IVDD [32] by maintaining the balance of mitochondrial dynamics. Western blot analysis revealed that Drp1 phosphorylation at serine 616 was increased in LPS-treated CEP cells (Fig. 3A, B). Consistently, LPS induced mitochondrial translocation of Drp1 (Fig. 3C-E). Confocal microscopy further confirmed the increased colocalization of phosphorylated Drp1 (green fluorescence) and MitoTracker (red fluorescence) (Fig. 3F). Nrf2-Exos and CAP-Nrf2-Exos effectively inhibited the excessive Drp1 activation (Fig. 3A-B) and mitochondrial translocation (Figure C-F) caused by LPS. Furthermore, CAP-Nrf2-Exos exerted better inhibitory effects on Drp1 than Nrf2-Exos.

**Fig. 4:**
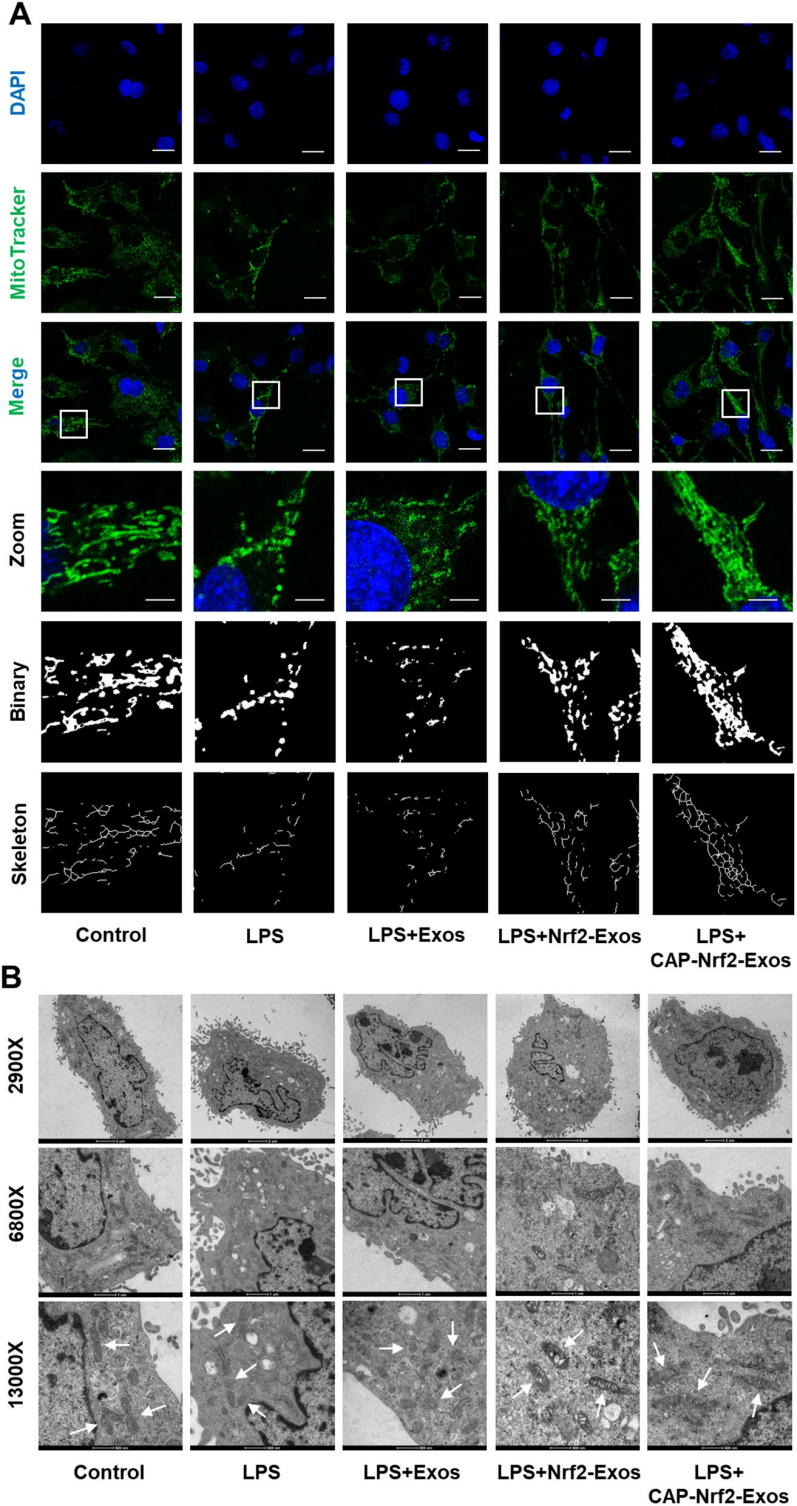
CAP-Nrf2-Exos improved mitochondrial morphology. (**A**) MitoTracker Green staining showing mitochondrial morphology in CEP cells after treatment with LPS and engineered exosomes. Scale bar, 20 μm and 5 μm. (**B**) The morphological ultrastructural appearance of mitochondria were observed by TEM. (*n* = 3, *p < 0.05; **p < 0.01; ***p < 0.001)

To observe the alterations in mitochondrial morphology, CEP cells were stained with MitoTracker Green. Images of mitochondria were captured by confocal microscopy and were processed and skeletonized by ImageJ software. The results clearly showed a mitochondrial network in the control group, and LPS shortened the lengths of the mitochondrial branches, leading to mitochondrial fragmentation (Fig. 4A). Control exosomes did not alter the increase in punctate or rod-shaped mitochondria in LPS-treated CEP cells. Interestingly, Nrf2-Exos and CAP-Nrf2-Exos dramatically prevented LPS-induced mitochondrial fragmentation and restored the mitochondrial network. Additionally, CAP-Nrf2-Exos exerted better therapeutic effects than Nrf2-Exos (Fig. 4A). TEM was used to further examine mitochondrial morphology. As shown in Fig. 4B, the impaired mitochondria in LPS-treated CEP cells were small and fragmented and had reduced or no cristae structures and disrupted membranes compared to healthy mitochondria. Treatment with Nrf2-Exos or CAP-Nrf2-Exos successfully maintained the relatively normal morphology of most mitochondria. Due to the used of chondrocyte-targeted engineering, CAP-Nrf2-Exos were even more effective than Nrf2-Exos at suppressing mitochondrial fragmentation.

### CAP-Nrf2-Exos protected CEP cells from mitochondrial dysfunction and apoptosis

**Fig. 5:**
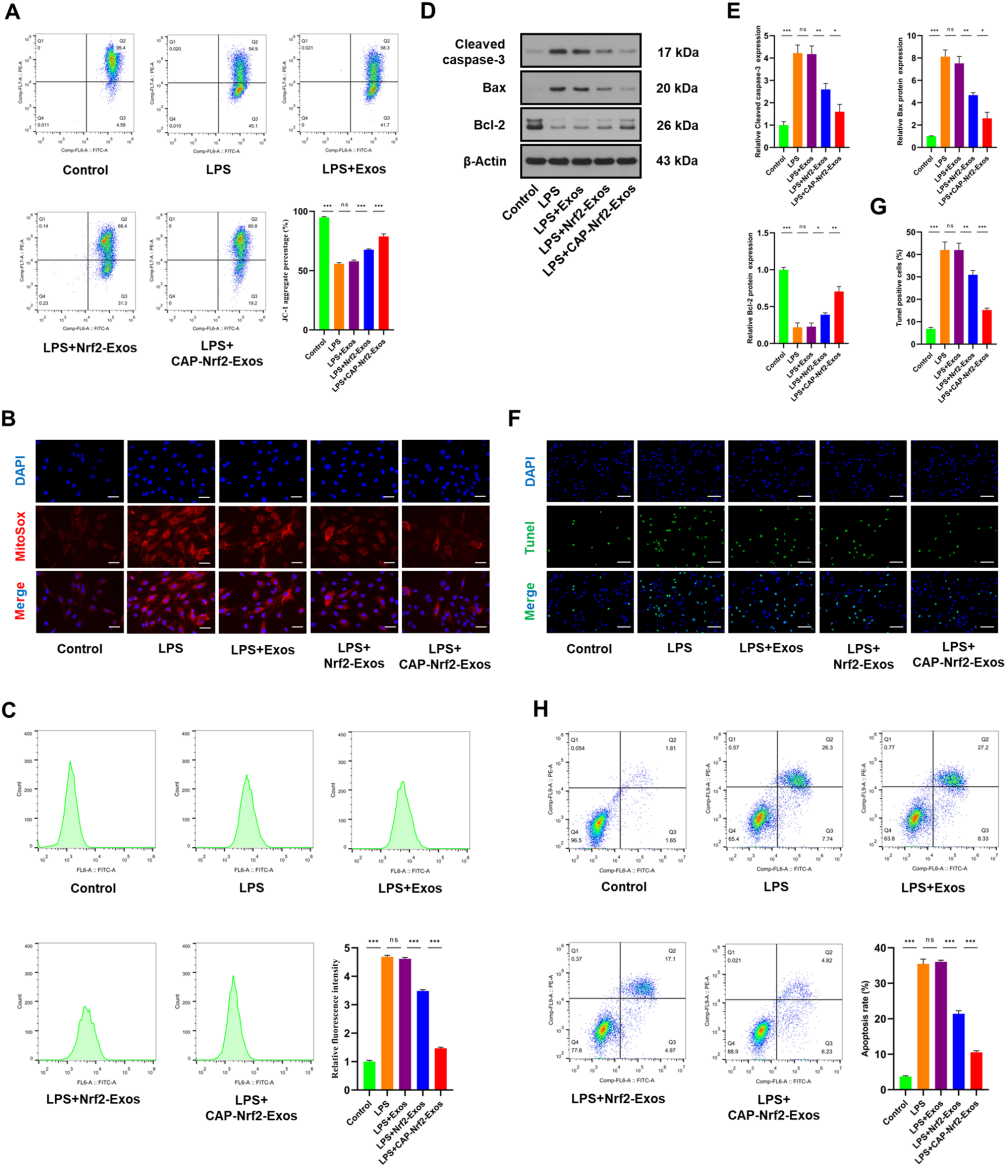
CAP-Nrf2-Exos alleviated mitochondrial dysfunction and apoptosis. (**A**) The MMP in CEP cells was assessed by flow cytometry using JC-1 staining. (**B**) The production of mitochondrial ROS in CEP cells was measured by MitoSOX Red staining. Scale bar, 50 μm. (**C**) The production of cellular ROS in CEP cells was detected by DCFH-DA staining and flow cytometry. (**D, E**) Western blot analysis and quantification of cleaved caspase-3, Bax, and Bcl-2 in CEP cells. (**F, G**) TUNEL staining showing the apoptosis rates of CEP cells. Scale bar, 100 μm (**H**) Flow cytometry with Annexin V-FITC/PI verifying the percentage of apoptotic CEP cells after treatment with LPS and engineered exosomes. (*n* = 3, *p < 0.05; **p < 0.01; ***p < 0.001)

Excessive mitochondrial fission leads to mitochondrial dysfunction and cell death. Flow cytometric analysis of JC-1 staining showed that LPS exacerbated the loss of mitochondrial membrane potential (MMP), while Nrf2-Exos and CAP-Nrf2-Exos exerted protective effects on mitochondria in LPS-treated CEP cells (Fig. 5A). MitoSOX Red and DCFH-DA staining revealed that LPS increased the production of mitochondrial and cellular ROS (Fig. 5B, C). In contrast, Nrf2-Exos or CAP-Nrf2-Exos ameliorated LPS-induced ROS accumulation. Apoptosis-related proteins were examined by Western blot analysis, and the results revealed increased levels of cleaved caspase-3 and Bax and decreased Bcl-2 expression in the LPS group; these effects could be reversed by Nrf2-Exos or CAP-Nrf2-Exos treatment (Fig. 5D, E). Terminal deoxynucleotidyl transferase biotin-dUTP nick end labelling (TUNEL) staining, Annexin V-FITC/PI staining and flow cytometry showed that LPS increased the number of apoptotic CEP cells, while Nrf2-Exos or CAP-Nrf2-Exos inhibited the proapoptotic effects of LPS (Fig. 5G-H). These data showed that the engineered exosomes protected mitochondria and exerted antiapoptotic effects. In addition, CAP-Nrf2-Exos exerted better therapeutic effects than Nrf2-Exos due to the presence of CAP.

### CAP-Nrf2-Exos attenuated cartilaginous endplate degeneration in vivo

**Fig. 6:**
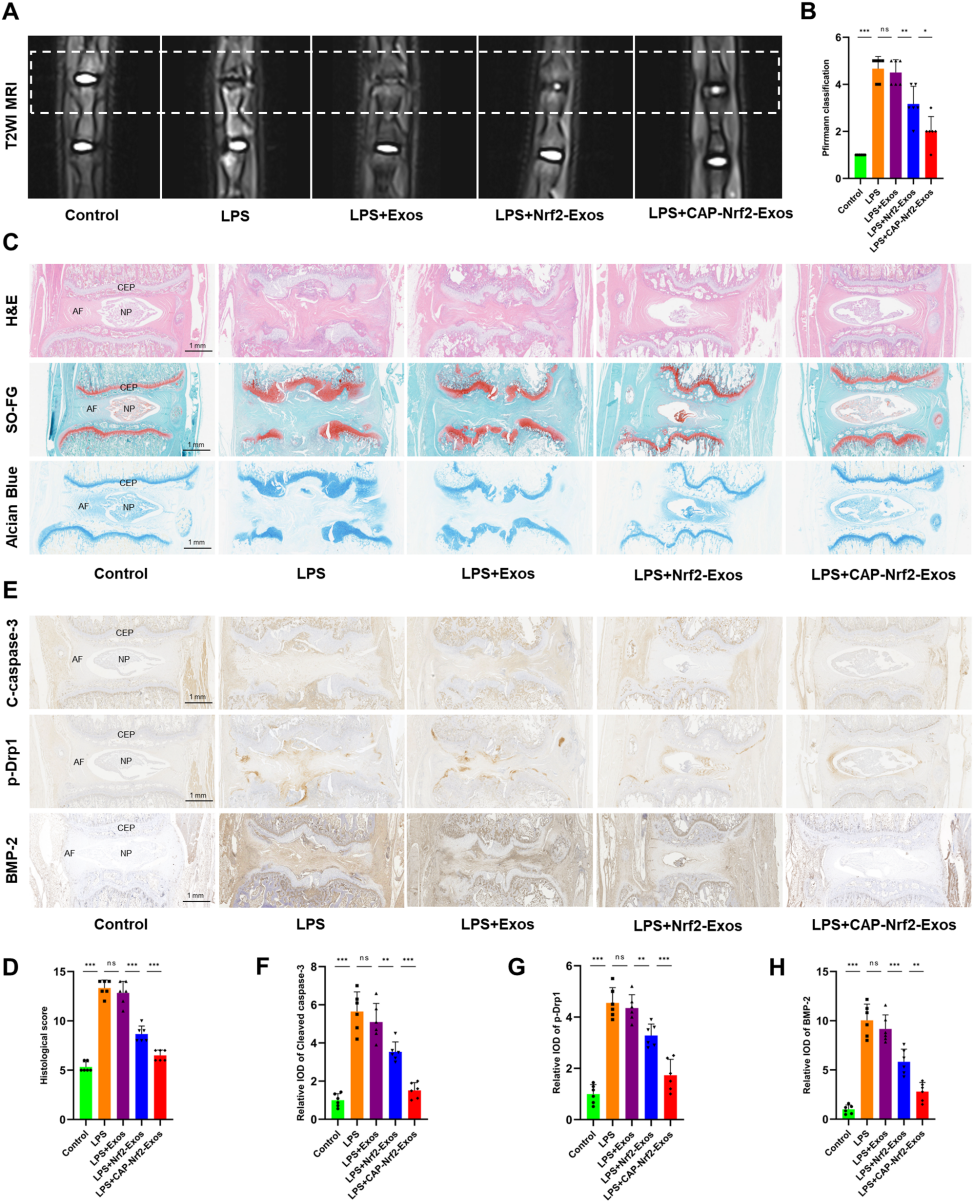
CAP-Nrf2-Exos attenuate cartilaginous endplate degeneration in vivo (**A**) The coccygeal vertebrae of rats were examined by MRI. (**B**) Evaluation of IVDD by Pfirrmann classification. (**C**) H&E staining, Safranin O-Fast Green staining and Alcian Blue staining of rat IVD samples. (**D**) Evaluation of IVDD by histological scoring. (**E-H**) Immunohistochemical analysis of cleaved caspase-3, p-Drp1 and BMP-2. (*n* = 6, *p < 0.05; **p < 0.01; ***p < 0.001)

We established a CEP degeneration rat model by subendplate injection of LPS. The rats were divided into control, LPS, LPS + Exos, LPS + Nrf2-Exos and LPS + CAP-Nrf2-Exos groups. After four weeks, the degenerative grade of IVDs was higher in the LPS group than in the control group, as shown by the Pfirrmann classification (Fig. 6A, B) and the histological score of IVDD (Fig. 6C, D). Histological analysis by H&E, Safranin O-Fast Green and Alcian Blue staining showed that LPS caused CEP degeneration, which was characterized by CEP damage and ossification and the loss of chondrocytes. Exos exerted no therapeutic effects, and Nrf2-Exos and CAP-Nrf2-Exos mediated repair. Surface modification endowed CAP-Nrf2-Exos with greater chondroprotective effects than Nrf2-Exos. Immunohistochemistry showed that LPS increased the expression of p-Drp1, cleaved caspase-3 and BMP-2 in IVD tissues (Fig. 6E-H). However, Nrf2-Exos and CAP-Nrf2-Exos reduced the expression of these factors. These results confirmed that engineered exosomes could delay the progression of inflammation-induced CEP degeneration in vivo.

## Discussion

Exosome engineering holds great promise for IVDD treatment. Research on the use of engineered exosomes for IVDD treatment is at an early stage. Liao et al. [33] showed that peroxiredoxin-2 (Prx2) was abundant in MSC-derived EVs (MSC-EVs) and exerted therapeutic effects on NP cells. Additionally, the caveolae-mediated endocytosis pathway was impaired in TNF-α-treated NP cells and caveolae-associated protein 2 (Cavin-2) was essential for EV uptake. Consequently, the researchers fused Lamp2b to Cavin-2 by a gene-editing technique, constructed a Cavin-2-Lamp2b lentiviral expression vector, transfected MSCs and isolated Cavin-2-modified MSC-EVs. The modified EVs restored the cellular uptake rate, delivered Prx2 into NP cells and inhibited TNF-α-induced pyroptosis. In another study, Tong et al. [34] investigated whether the delivery efficiency of MSC-EVs was decreased in NP cells under hypoxic conditions because of an increase in endocytic recycling. Hypoxia inducible factor-1 (HIF-1)-induced upregulation of Rab-coupling protein (RCP), which is also known as RAB11 family interacting protein 1 (RAB11FIP1), promoted the Rab11a-dependent recycling of internalized MSC-EVs by facilitating the interaction between Rab11a and MSC-EVs. To inhibit endocytic recycling and increase cargo delivery efficiency under hypoxic conditions, the researchers loaded si-RCP into MSC-EVs by electroporation to generate engineered exosomes. The engineered MSC-EVs loaded with si-RCP exerted better regenerative effects on IVDD than natural EVs in vitro and in vivo. Luo et al. [35] extracted CEP stem cell-derived exosomes (CESC-Exos) and found that CESC-Exos migrated from the annulus fibrosus to the NP and improved puncture-induced IVDD progression. Mechanistically, CESC-Exos could transport Sphk2, which is a regulator of autophagy and senescence, to NP cells and activate the PI3K/AKT signalling pathway. To increase the therapeutic efficacy, Sphk2 was overexpressed in CESCs by lentiviruses (Lenti-Sphk2-CESCs), and engineered exosomes isolated from CESCs (Lenti-Sphk2-Exos) were enriched in Sphk2. Furthermore, the researchers modified hydrogels with the extracellular matrix of costal cartilage (ECM-Gels) and loaded the hydrogels with Lenti-Sphk2-CESCs. The hydrogels were injected near the CEP of rats, and Lenti-Sphk2-Exos carrying increased Sphk2 were stably released into NP cells, thereby increasing autophagy and suppressing NP cell senescence. Although these researchers successfully improved the efficacy of exosome-based IVDD treatment, the researchers used surface modification or cargo loading alone. To realize the full potential of engineered exosomes, we modified exosomes with CAP peptide on the surface to target chondrocytes and loaded Nrf2 into the exosomes by an endogenous method. Our results showed that this engineering strategy improved chondrocyte targeting by CAP-Nrf2-Exos and enhanced chondroprotective effects. In addition, IVDD is a complex and multifactorial process. Most studies have focused on NP cells. However, CEP cells are also involved in the pathogenesis of IVDD. We successfully generated novel CEP chondrocyte-targeted exosomes enriched in Nrf2, and CAP-Nrf2-Exos exerted therapeutic effects against CEP degeneration and IVDD.

Transfecting donor cells with overexpression vectors is a feasible approach for loading bioactive molecules into exosomes. Li et al. [36] found that exosomes secreted by adipose-derived stem cells (ADSC-Exos) could ameliorate ROS accumulation, inflammatory cytokine expression and senescence in endothelial progenitor cells (EPCs) under high-sugar conditions. Researchers constructed an Nrf2 overexpression plasmid and transfected it into ADSCs. Nrf2 protein levels were increased in exosomes isolated from Nrf2-overexpressing ADSCs. Accordingly, the modified exosomes showed better positive effects than natural exosomes on glucose-induced EPCs and a diabetic foot ulcer rat model. Yang et al. [16] engineered exosomes by cotransfecting the RVG-Lamp2b plasmid and NGF overexpression plasmid into HEK293T cells. Exosomes secreted by the transfected cells (NGF@ExoRVG) efficiently transported NGF mRNA and protein to the ischaemic region in a photothrombotic ischemia rat model. NGF@ExoRVG exerted neuroprotective effects by modulating microglial polarization, inhibiting apoptosis and promoting neurogenesis. Sun et al. [37] transfected a lentivirus containing a hypoxia inducible factor 1-alpha (HIF-1α)-overexpression vector into MSCs. HIF-1α expression levels were increased in exosomes isolated from modified MSCs (HIF-1α-Exos). Treatment of human umbilical vein endothelial cells (HUVECs) with HIF-1α-Exos increased angiogenesis, migration and proliferation under hypoxic conditions. In addition, HIF-1α-Exos preserved heart function by increasing angiogenesis and reducing fibrosis in a rat myocardial infarction model. Gonzalez-King et al. [38] reported that exosome secretion was increased in HIF-1α-overexpressing donor MSCs. The Notch ligand Jagged1 was incorporated into exosomes isolated from MSCs after HIF-1α was overexpressed (Jagged1-Exos). Jagged1-Exos triggered the Notch signalling pathway and induced angiogenesis in HUVECs. Subcutaneous injection of Jagged1-Exos promoted angiogenesis, as shown by the Matrigel plug assay. The present study aimed to explore the feasibility and effectiveness of loading engineered exosomes with Nrf2, which is the central regulator of antioxidant responses in CEP cells. Our data confirmed that Nrf2-loaded engineered exosomes increased Nrf2 levels and promoted Nrf2 nuclear translocation in recipient cells.

Genetically fusing cell-targeted peptides to exosome membrane proteins is commonly used to alter the targeting of exosomes. Delivering target genes through cartilage into chondrocytes is challenging due to the lack of vascularity and dense extracellular matrix. Pi et al. [29] identified chondrocyte-affinity peptide (CAP) by phage display technology. Surprisingly, this peptide could specifically and efficiently interact with chondrocytes without any species specificity. Liang et al. [39] fused CAP to the Lamp2b protein on the exosome surface and loaded miR-140 into the exosomes by electroporation. The researchers chose dendritic cells as donors and acquired engineered exosomes (CAP-Exos/miR-140). CAP-Exos/miR-140 could specifically enter chondrocytes and deliver miR-140. CAP-Exos/miR-140 treatment downregulated the expression of ECM catabolism-related proteins in IL-1β-induced chondrocytes. After intra-articular injection in an osteoarthritis rat model, CAP-Exos/miR-140 remained in the joints rather than diffusing to other organs, thereby facilitating cartilage penetration of the exosomes to the middle zone through the dense ECM and alleviating OA progression. In another study by Liang et al. [40], the researchers fused CAP-Exos with liposomes and created a hybrid exosome (hybrid CAP-Exos) that encapsulated CRISPR/Cas9 plasmids. The hybrid CAP-Exos penetrated the deep region of the cartilage ECM, transported the Cas9 sgMMP-13 plasmid to chondrocytes, efficiently knocked down the MMP-13 gene and improved ECM metabolism in an OA rat model. Our results verified that CAP facilitated the interaction between exosomes and the CEP in vitro and in vivo. In addition to articular cartilage, the CAP can also target the cartilaginous endplate, indicating its potential use in IVDD treatment.

Despite the ability of CAP-Nrf2-Exos to suppress mitochondrial fission in CEP cells and delay CEP degeneration in vivo, there are several limitations in this study. First, repeated administration may increase the risk of anaesthetic complications and surgical site infections. To increase the retention and therapeutic potential of exosomes, engineered exosomes can be combined with biomaterials such as hydrogels and scaffolds. Second, we chose HEK293T cells as exosome donor cells to better investigate the biological effects of Nrf2 and CAP on CEP cells. However, stem cell-derived exosomes may exert stronger therapeutic effects because they carry many endogenous cytoprotective molecules. Third, in addition to transfection, there are many other cargo loading techniques. Each method has its own advantages and disadvantages and it may be difficult to determine the best method. To avoid the drawback of canonical intradiscal injection, which might cause additional physical damage to IVDs, a subendplate injection technique was used to administer the engineered exosomes to rat coccygeal vertebrae. We hypothesize that transpedicular injection could be a safe route for administering exosome-based IVDD therapy in the clinic. However, additional studies are needed to determine whether this approach is feasible.

## Conclusions

In summary, we successfully performed chondrocyte-targeted delivery of Nrf2 to CEP cells and cartilaginous endplates by engineering CAP-Nrf2-Exos. CAP-Nrf2-Exos increased Nrf2 expression, activated Nrf2 nuclear translocation and enhanced the endogenous antioxidant defence system in CEP cells under inflammatory conditions. Mechanistically, CAP-Nrf2-Exos inhibited Drp1 phosphorylation and mitochondrial translocation. Moreover, excessive Drp1-mediated mitochondrial fission and aberrant mitochondrial morphology and function were rescued by CAP-Nrf2-Exos. The engineered exosomes protected CEP cells against apoptosis through the Nrf2-ROS-Drp1 axis. In vivo assays further confirmed that subendplate injection of CAP-Nrf2-Exos ameliorated LPS-induced CEP degeneration in a rat model.

## Materials and methods

### Ethical statement

All experiments involving animals were approved by the Animal Care and Use Committee of Fudan University (No. 202,309,012 S).

### Vector construction and transfection

The lentiviral Nrf2 expression vector and the CAP-Lamp2b plasmid were constructed by GeneChem (Shanghai, China). To obtain engineered exosomes, HEK293T cells (Procell, China) were transfected with the Nrf2-overexpression lentivirus, and stable strains were selected with neomycin. Then, HEK293T cells overexpressing Nrf2 were further transfected with the CAP-Lamp2b plasmid using Lipofectamine 2000 (Invitrogen, USA) according to the manufacturer’s instructions. CAP-Nrf2-Exos were isolated from the supernatant of HEK293T cells that were cotransfected with the Nrf2 expression lentivirus and the CAP-Lamp2b plasmid by differential centrifugation. Natural exosomes were also collected from HEK293T cells that were not transfected with any lentiviruses or plasmids for use as controls.

### Exosome isolation and purification

HEK293T cells were cultured in Dulbecco’s modified Eagle’s medium (DMEM; PM150210, Procell, China) supplemented with 10% foetal bovine serum (FBS; 164,210, Procell, China) and 1% penicillin‒streptomycin (PB180120, Procell, China). Additionally, bovine extracellular vesicles in FBS were depleted by ultracentrifugation at 200,000 × g for 6 h at 4 °C. The cells were kept in a humidified incubator (5% CO_2_, 37 °C). For exosome isolation, the supernatant was centrifuged first at 300 × g for 10 min, then at 3000 × g for 15 min, and at 10,000 × g for 1 h at 4 °C. After being filtered through a 0.22 μm filter (Millipore, USA), the filtrate was centrifuged at 200,000 × g for 90 min at 4 °C. Finally, the pellet was resuspended in PBS for further experiments.

### Exosome identification

For morphological analysis, exosomes were fixed with 2.5% glutaraldehyde and placed on carbon-coated copper grids for 5 min. The sample was then stained with 2% phosphotungstic acid (Sigma‒Aldrich, USA) for 3 min. Images were captured by transmission electron microscopy (TEM; HT7700, Hitachi, Japan). Exosome number and size were assessed by nanoparticle tracking analysis (NTA) using a ZetaView PMX 110 (Particle Metrix, Germany). The surface markers of the exosomes were verified by Western blot analysis. The following antibodies were used: anti-CD9 (1:1000, AF5139, Affinity Biosciences), anti-CD63 (1:1000, PA5-92370, Thermo Fisher Scientific), anti-TSG101 (1:1000, ab125011, Abcam) and anti-Lamp2b (1:500, ab18529, Abcam).

### Rat CEP cell culture and treatment

Isolation of primary CEP cells was performed according to the protocol reported by Piprode et al. [41]. Thirty-five Sprague-Dawley rats (male, 8 weeks old) were used to extract primary CEP cells. The CEP tissues were excised from the thoracic and lumbar vertebrae (T1-T13 and L1-L6). The CEP tissues were fragmented and digested with 0.2 mg/ml type II collagenase (17,101,015, Thermo Fisher Scientific) for 12 h. The released cells were collected by centrifugation for 5 min at 500 × g. The CEP cells were then resuspended in DMEM supplemented with 10% FBS and maintained in a humidified incubator at 37 °C with 5% CO_2_. The culture medium was changed twice per week. Approximately, 1 × 10^5^ CEP cells were obtained from each rat. The second or third passage of CEP cells were used for further experiments. To mimic CEP degeneration in vitro, CEP cells were treated with 10 µg/mL LPS (BS904, Biosharp, China) for 24 h. Then, the CEP cells were incubated with 100 µg/mL Exos, CAP-Exos, Nrf2-Exos or CAP-Nrf2-Exos for 24 h to investigate the therapeutic effects of the engineered exosomes and the underlying mechanisms [33].

### Exosome labelling and visualization

The isolated exosomes were stained with PKH26 (MX4021, MKBio, China) for 5 min at room temperature. The exosomes were separated from the unincorporated dye by centrifugation at 120,000 × g for 90 min and washed twice with PBS. After being purified, the labelled exosomes were resuspended in medium and incubated with CEP cells for internalization assays. The nuclei of CEP cells were stained with 4’,6-diamidino-2-phenylindole (DAPI; C1006, Beyotime, China). The uptake of labelled exosomes by CEP cells was evaluated by fluorescence microscopy (IX51, Olympus, Japan).

### Quantitative real-time PCR (qRT‒PCR)

Total RNA was extracted from cells using TRIpure reagent (EP013, ELK Biotechnology) according to the manufacturer’s instructions. The following primers were used: rat Nfe2l2 (Gene ID: 83619) (F: 5’-TCCTCTGCTGCCATTAGTCA-3’, R: 5’-GTGCCTTCAGTGTGCTTCTG-3’) and rat Actb (Gene ID: 81,822) (F: 5’- TTCGTTGCCGGTCCACACCC-3’, R: 5’- GCTTTGCACATGCCGGAGCC-3’). β-Actin was used for normalization.

### Western blot analysis

The cells were lysed on ice in buffer containing protease inhibitors (AS1008, Aspen). The protein fractions were collected and separated by sodium dodecyl sulfate‒polyacrylamide gel electrophoresis (SDS‒PAGE). Then, the proteins were electrophoretically transferred onto polyvinylidene fluoride (PVDF) membranes (IPVH00010, Millipore). The membranes were blocked with 5% skim milk. Then, primary antibodies were added and incubated overnight at 4 °C. The membranes were then incubated with horseradish peroxidase-conjugated secondary antibodies. The antibodies used in this study included anti-Nrf2 (1:1000, PA5-27882, Thermo Fisher Scientific), anti-β-Actin (1:10,000, TDY051, TDY Biotech), anti-Histone H3 (1:10,000, #4499, Cell Signaling Technology), anti-Drp1 (1:1000, #8570, Cell Signaling Technology), anti-p-Drp1 (Ser616) (1:500, PA5-106169, Thermo Fisher Scientific), anti-VDAC1 (1:3000, ab15895, Abcam), anti-NOQ1 (1:3000, ab80588, Abcam), anti-HO-1 (1:5000, 10701-1-AP, Proteintech Group), anti-SOD2 (1:3000, ab68155, Abcam), anti-cleaved Caspase-3 (1:1000, AF7022, Affinity Biosciences), anti-Bax (1:2000, #2772, Cell Signaling Technology) and anti-Bcl-2 (1:1000, ab19649, Abcam).

### Immunofluorescence (IF) staining

CEP cells were fixed with 4% paraformaldehyde for 20 min and then permeabilized with 0.5% Triton X-100 for 20 min. After being blocked with 5% bovine serum albumin, the samples were incubated overnight with primary antibodies, against anti-Nrf2 (1:300, 16396-1-AP; Proteintech Group) and p-Drp1 (1:200, PA5-106169; Thermo Fisher Scientific). After being washed with PBS three times, the samples were incubated with the corresponding secondary antibodies for 40 min in the dark. The nuclei were stained with DAPI (C1006; Beyotime, China) for 20 min. Finally, images were captured with a fluorescence microscope (IX51; Olympus, Japan) and fluorescence confocal microscope (LSM880; Zeiss, Germany).

### Apoptosis assay

Flow cytometry and Annexin V-FITC/PI were used to examine CEP cell apoptosis. An Apoptosis Analysis Kit (AO2001-02P-H, Sungene, Biotech) was used to stain CEP cells according to the manufacturer’s instructions. Briefly, CEP cells were incubated with 5 µL of Annexin V-FITC for 10 min in the dark after being resuspended. Then, the CEP cells were incubated with 5 µL of PI for 5 min in the dark. Finally, apoptosis was measured by flow cytometry (CytoFLEX, Beckman Coulter, USA).

DNA damage in CEP cells was evaluated by terminal deoxynucleotidyl transferase biotin-dUTP nick end labelling (TUNEL) staining. CEP cells were fixed and stained with a TUNEL Cell Apoptosis Detection Kit (G1504, Servicebio Biological) according to the manufacturer’s instructions, and the nuclei were stained with DAPI.

### Mitochondrial membrane potential (MMP) assessment

A JC-1 Assay Kit (C2006, Beyotime) was used to measure MMP. CEP cells were incubated with JC-1 working solution for 20 min at 37 °C. Then, the samples were centrifuged at 300 × g at 4 °C for 3 min and washed twice with JC-1 buffer solution. After being resuspended, the stained CEP cells were analysed by flow cytometry. The MMP of CEP cells was assessed by determining the ratio of JC-1 aggregates to monomers.

### Measurement of mitochondrial reactive oxygen species (mtROS) and cellular ROS

Cellular ROS and mtROS were examined using an ROS Assay Kit (S0033, Beyotime) and MitoSOX Red (M36008, Thermo Fisher Scientific), respectively. CEP cells were incubated with MitoSOX Red for 10 min or DCFH-DA for 20 min at 37 °C in the dark. The cells were then washed three times with PBS. The fluorescence intensity was measured by flow cytometry (CytoFLEX, Beckman Coulter, USA).

### MitoTracker staining

MitoTracker Green (C1048, Beyotime) and MitoTracker Red CMXRos (C1049B, Beyotime) were used to stain mitochondria according to the manufacturer’s instructions. Briefly, the media was discarded. The cells were incubated with MitoTracker Green working solution for 30 min or with MitoTracker Red working solution at 37 °C for 20 min. Images were captured under a fluorescence confocal microscope (LSM880, Zeiss, Germany).

### Transmission electron microscopy

CEP cells were collected by trypsinization and washed with PBS. The cell pellets were fixed with 2.5% glutaraldehyde overnight at 4 °C. Then, the cells were postfixed with 1% osmium tetroxide for 2 h at 37 °C. The pellets were subsequently dehydrated and embedded in Epon 812. Ultrathin sections were stained with uranyl acetate and lead citrate. Images were obtained by TEM (HT7700, Hitachi, Japan).

### Establishment of a rat model of CEP degeneration

Thirty Sprague‒Dawley rats (male, 8 weeks old) were randomly divided into five groups: the control group, LPS group, LPS + Exo group, LPS + Nrf2-Exo group and LPS + CAP-Nrf2-Exo group. The rats were anaesthetized by an intraperitoneal injection of 40 mg/kg pentobarbital. A rat model of CEP degeneration was established by subendplate injection of 20 µL of LPS (10 µg/mL), and the control group was treated with the same volume of PBS. Briefly, Co7/8 was located by digital palpation, and the rats were subjected to coccygeal vertebral drilling through the cortical bone and into the bone marrow percutaneously. Bone drilling was manually performed on the 8th coccygeal vertebral body near the Co7/8 disc (subendplate) by using a 22G lumbar puncture needle. Then, a tunnel was created, and subendplate injection into the vertebral bone marrow was accomplished by using a microlitre syringe. To investigate the therapeutic effects of exosomes, 20 µL of Exos (100 µg/mL) was injected into the rats in the LPS + Exo group, 20 µL of Nrf2-Exos (100 µg/mL) was injected into the rats in the LPS + Nrf2-Exo group, and 20 µL of CAP-Nrf2-Exos (100 µg/mL) was injected into the rats in the LPS + CAP-Nrf2-Exo group once per week for four weeks; rats in the control and LPS groups were treated with the same amount of PBS. Finally, the animals were returned to their cages with unrestricted activity.

### In vivo imaging assay

Exosomes were labelled with DiR iodide (40757ES25; Yeasen Biotechnology) according to the manufacturer’s protocols. The rats were treated with the labelled exosomes via subendplate injection using a microlitre syringe. At each time point, images were captured with a small animal imaging system (UVP iBox Scientia, Analytik Jena, Germany).

### MRI

The coccygeal vertebrae of the rats were examined by MRI (BioSpec 70/30 USR, Bruker, Germany) to assess the signal and structural changes in the sagittal T2-weighted images. The degree of IVDD was evaluated using the Pfirrmann grading system [42].

### Histological staining and immunohistochemistry

The samples were collected, fixed, decalcified, dehydrated, embedded in paraffin, and cut into sections. H&E staining, Safranin O-fast green staining and Alcian blue staining were performed to assess the degree of CEP degeneration and IVDD [31]. Immunohistochemistry was performed to analyse the expression levels of cleaved caspase-3 (1:200, AF7022, Affinity Biosciences), BMP-2 (1:200, PA5-85956, Thermo Fisher Scientific) and p-Drp1 (1:500, PA5-106169, Thermo Fisher Scientific). The integrated optical density (IOD) of the immunohistochemical images was quantified using ImageJ software.

### Statistical analysis

The results are presented as the mean ± standard deviation. *n* = 3 biological replicates were performed for in vitro experiments and *n* = 6 biological replicates were performed for in vivo experiments. The statistical analyses were performed using SPSS 21.0 and GraphPad Prism 9.0 software. The data were analysed using Student’s t test for comparisons between two groups and one-way analysis of variance followed by Tukey’s test for comparisons among multiple groups. A value of *p* < 0.05 indicated statistical significance (*p < 0.05, ** *p* < 0.01 or ***p < 0.001).

## Data Availability

No datasets were generated or analysed during the current study.

## Abbreviations

CEP: Cartilaginous endplate
IVDD: Intervertebral disc degeneration
Drp1: Dynamin-related protein 1
CAP: Chondrocyte-affinity peptide
Nrf2: Nuclear factor E2-related factor 2
MCs: Modic changes
MRI: Magnetic resonance imaging
NP: Nucleus pulposus
AF: Annulus fibrosus
ROS: Reactive oxygen species
Keap1: Kelch-like ECH-associated protein 1
ARE: Antioxidant response element
sMAF: Small musculoaponeurotic fibrosarcoma
NQO1: NADP(H) quinone oxidoreductase 1
HO-1: Haem-oxygenase-1
SOD2: Superoxide dismutase 2
Exos: Exosomes
TEM: Transmission electron microscopy
NTA: Nanoparticle tracking analysis
TUNEL: Terminal deoxynucleotidyl transferase biotin-dUTP nick end labelling
